# Supplementary material for: First Report of the Plasmid-mediated fosB Gene in Enterococcus faecalis from Pigs
Source: Genes (Basel). 2021 Oct 23;12(11):1684. doi: 10.3390/genes12111684 (PMC8624268; doi:10.3390/genes12111684)
Supplement: Supplementary file 1 [file genes-12-01684-s001.zip › Supplementary data.pdf]

Supplementary material

Table S1 primers used in this study.

Gene	Sequence	Amplicon size (bp)	reference
fosA	F: GCTGCACGCCCGCTGGAATA	217	Zhang et al., 2020
	R: -CGACGCCCCCTCGCTTTTGT		
fosB	F: CAGAGATATTTTAGGGGCTGACA	312	Zhang et al., 2020
	R: CTCAATCTATCTTCTAAACTTCCTG		
fosC	F: GGGTTACATGCCCTTGCTCA	354	Zhang et al., 2020
	R: AACCCGCACAACGACAGATG		
fosX	F: ATGATCAGTCATATGACATTTATCG	243	Zhang et al., 2020
	R: ATTTAGCCCCTTGTCGATAACG		

Zhang, X., Bi, W., Chen, L., Zhang, Y., Fang, R., Cao, J., Zhou, T., 2020, Molecular mechanisms and epidemiology of fosfomycin resistance in enterococci isolated from patients at a teaching hospital in China, 2013-2016. J Glob Antimicrob Resist 20, 191-196.

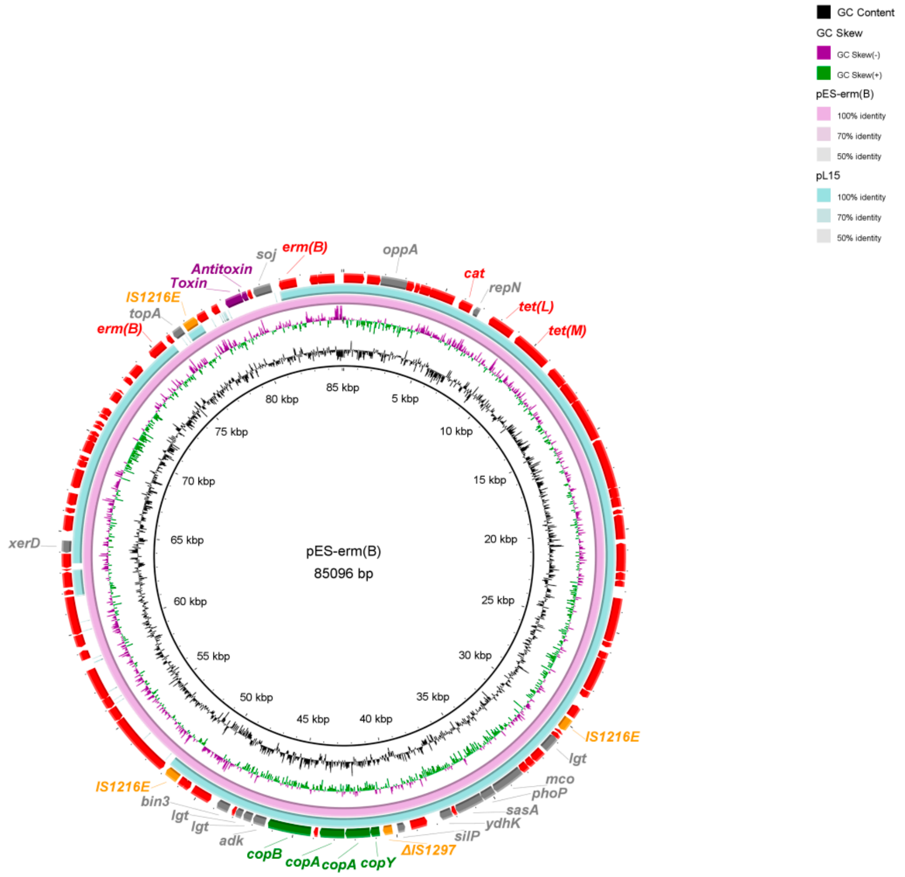

Fig S1. The genetic contents of plasmid pES-erm(B). Circular representation of alignments between reference *erm*(B) carrying plasmid pL15 (accession no. CP042214.1) and the homologous plasmid from *E. faecalis* ES1. The alignments were generated by Blastn and visualized by BLAST Ring Image Generator.

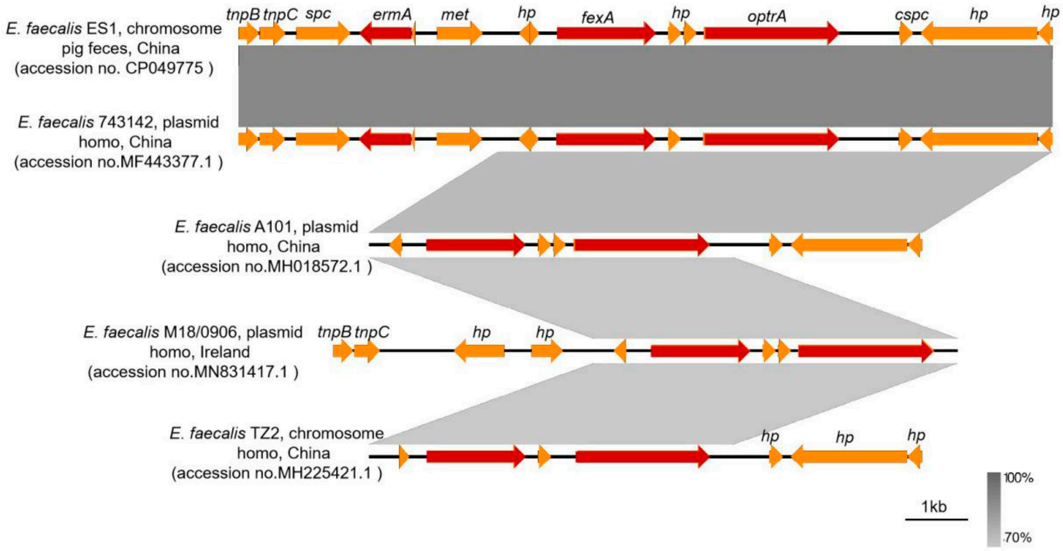

Fig S2. Genetic environment of *optrA*. Schematic representation and comparison of the genetic environment of the *optrA*-flanking region from this study and previous reported. Arrows indicate the direction of transcription of each of the genes, and different genes are shown in different colors. Regions of ≥70.0% nucleotide sequence identity are shaded in grey.
